# Attitudes of people with inherited retinal conditions toward gene editing technology

**DOI:** 10.1002/mgg3.803

**Published:** 2019-06-12

**Authors:** Lily Hoffman‐Andrews, Ronit Mazzoni, Michelle Pacione, Rosemarie Garland‐Thomson, Kelly E. Ormond

**Affiliations:** ^1^ Department of Genetics Stanford University School of Medicine Stanford CA; ^2^ Department of Genetics Santa Clara Valley Medical Center San Jose CA; ^3^ Department of English Emory University Atlanta GA; ^4^ Stanford Center for Biomedical Ethics Stanford University School of Medicine Stanford CA; ^5^ Present address: Penn Center for Inherited Cardiac Disease Penn Medicine Philadelphia PA; ^6^ Present address: Center for Fetal Medicine and Reproductive Genetics, Brigham & Women’s Hospital Boston MA

**Keywords:** disability, gene editing, gene therapy, Leber congenital amaurosis, retinitis pigmentosa

## Abstract

**Background:**

The views of people with genetic conditions are crucial to include in public dialogue around developing gene editing technologies. This qualitative study sought to characterize the attitudes of people with inherited retinal conditions (retinitis pigmentosa [RP] and Leber congenital amaurosis [LCA]) toward gene editing.

**Methods:**

Individuals with RP (*N* = 9) and LCA (*N* = 8) participated in semi‐structured qualitative interviews about their experience with and attitudes toward blindness, and their views about gene editing technology for somatic, germline, and enhancement applications.

**Results:**

Participants saw potential benefits from gene editing in general, but views about its use for retinal conditions varied and were influenced by personal perspectives on blindness. Those who felt more negatively toward blindness, particularly those with later onset blindness, were more supportive of gene editing for retinal conditions. Concerns about both germline and somatic editing included: the importance of informed consent; impacts of gene editing on social attitudes and barriers affecting blind people; and worries about “eliminating” blindness or other traits.

**Conclusion:**

People with RP and LCA have diverse attitudes toward gene editing technology informed by their own lived experience with disability, and many have concerns about how the ways in which it is discussed and implemented might affect them.

## 1 INTRODUCTION

Recent advances in gene editing technology, particularly the efficient and relatively inexpensive CRISPR‐Cas9 system, have brought gene editing to the forefront of medical research as well as bioethical debate. The first clinical trials using somatic gene editing technology are already underway (Kaiser, 2017), as are early forays into gene editing in embryos (Cyranoski & Ledford, 2018; Ma et al., 2017; Zeng et al., 2018). However, ethical concerns about uses of this technology remain, and many have called for the voices of those who stand to be most impacted by its development, people with genetic conditions and disabilities, to be included in the conversation (Check Hayden, 2016; Shakespeare, 2015).

Many of the challenging disability‐related questions arising from gene editing, particularly in germline applications, overlap with those surrounding prenatal genetic testing, which has been controversial among disability advocates who are troubled by the use of this technology to prevent the birth of disabled children (Parens & Asch, 2003). Studies of views toward prenatal testing among people with genetic conditions and disabilities have found them to vary both between and within disability groups (e.g. Ahmed et al., 2015; Barlevy, Wasserman, Stolerman, Erskine, & Dolan, 2012; Chen & Schiffman, 2000; Middleton, Hewison, & Mueller, 1998). These attitudes are influenced by personal attitudes about and experiences with disability, as well as disability identity and involvement with disability culture and community^1^ (Boardman & Hale, 2018; Boardman, Young, & Griffiths, 2018; Gollust, Thompson, Gooding, & Biesecker, 2003; Roadhouse et al., 2018; Stern et al., 2002).

While somatic gene editing or gene therapy, which could potentially treat or cure genetic conditions or disabilities, has generally been seen as less controversial than germline editing by bioethicists, many in the disability community have also raised issues regarding it and other “curative” technologies (Clare, 2017; Hahn & Belt, 2004). For example, many Deaf people have concerns about the use of cochlear implants in deaf infants and children on the grounds that it violates their autonomy and threatens the continuation of Deaf culture (Crouch, 1997; Most, Wiesel, & Blitzer, 2007). Many autistic people who identify with the neurodiversity movement have also opposed the search for “cures” of what they consider a constitutive aspect of their selves and identities (Bagatell, 2010; Kapp, Gillespie‐Lynch, Sherman, & Hutman, 2013).

Another controversial issue surrounding gene editing and gene therapy is the potential use of these technologies for “enhancement” above species‐typical functioning. In the bioethical literature, therapeutic uses of gene therapy have been traditionally regarded as less controversial and “enhancement” uses as more troubling (Anderson, 1985). Some have questioned, however, whether the line between therapy and enhancement can be so clearly drawn in practice, and have pointed out the potentially troubling implications of how “normal” must be defined in drawing such a line (Scully & Rehmann‐Sutter, 2001).

Despite this extensive theoretical discussion, very little research has been conducted on the attitudes of people with disabilities toward either somatic or germline gene editing. One study looking at the attitudes of medical professionals and people with disabilities toward somatic gene therapy found individuals with disabilities raised a broader swath of ethical concerns than professionals, including issues surrounding identity and the positive value of disability (Scully, Rippberger, & Rehmann‐Sutter, 2004). People with disabilities are uniquely situated to perceive ethical and social dimensions of genetic technology that nondisabled people, including scientists and medical professionals, cannot (Patterson & Satz, 2002). It is thus vitally important that the views of those who stand to be most affected by the development of gene editing technology, people with genetic conditions and disabilities, are documented and considered as policies and norms around these technologies are developed.

Retinitis pigmentosa (RP) and Leber congenital amaurosis (LCA) are two inherited forms of retinal dystrophy that can cause vision loss and blindness. Visual impairment due to LCA generally presents in infancy, while the onset of RP ranges from childhood to adulthood. These conditions have been early targets of research in gene therapy and gene editing; the FDA approved the first‐ever gene therapy for one form of LCA in 2017 (FDA, 2017), and clinical trials of a CRISPR‐based therapy for another form of LCA are set to begin in the near future (Sheridan, 2018). CRISPR‐Cas9 has also been demonstrated to be effective in animal models of RP (Bakondi et al., 2016; Zhu et al., 2017). The aim of this study was to explore the views of people with these conditions toward gene editing for somatic, germline, and enhancement applications, both related to these conditions and more generally, and how these attitudes are informed by their experiences with and attitudes toward blindness.

## 2 METHODS

### 2.1 Ethical compliance

This research was approved by the Stanford University Institutional Review Board. All participants provided oral consent to participate and were provided with a $20 gift card as incentive for participation.

### 2.2 Subjects

Participants who identified as having a diagnosis of LCA or RP and were over the age of 18 were recruited through the e‐mail listserv for the National Federation of the Blind (NFB), a national organization of people who are blind or have low vision (7,500 subscribers). Recruitment was approved by the NFB's research committee. Individuals interested in participating were asked to complete a demographic screening survey administered through REDCAP software hosted at Stanford University (Harris et al., 2009). The survey contained questions about demographics as well as diagnosis, age of onset, whether they had a family history of the condition, and whether they had children. Stratified purposive sampling (Patton, 2002) was used to select a demographically diverse sample and allow for comparison between participants with different conditions and ages of onset.

### 2.3 Method

A single investigator (LHA) completed a 30‐75‐min (mean length: 56 min) semi‐structured telephone interview that included questions about participants’ experiences with and attitudes toward blindness, and their attitudes toward somatic, germline, and enhancement uses of gene editing technology, after being given a brief description of the technology and these potential uses. The interview guide (Appendix I) was developed by three of the investigators (LHA, RM, KEO—all genetic counselors with qualitative research experience and one with involvement in the blind community) after a review of the bioethics literature about gene therapy and gene editing as well as disability studies literature.

Interviews were audio recorded and transcribed, with the exception of one interview for which audio recording failed; this interview was analyzed based on detailed notes. Transcripts were then inductively coded using Dedoose software (SocioCultural Research Consultants, 2018) by three members of the research team (LHA, MP, KEO, all genetic counselors with qualitative research experience). These investigators discussed and adjudicated codes after the first round of coding. Inter‐rater reliability was calculated on a subset of transcripts by LHA and MP with a pooled Cohen's kappa 0.77, indicating substantial agreement (Landis & Koch, 1977). A single investigator (LHA) then systematically coded the remaining transcripts and developed a preliminary list of themes based on commonalities emerging from multiple interviews. KEO and RM (a scholar with expertise in bioethics and disability studies) gave feedback on the preliminary themes, which were then revised to create a final list of themes. A summary of these themes was sent to participants before finalizing the analysis to give them the opportunity to provide feedback (Patton, 1999). The themes selected were present in at least 50% of interviews. Quotes presented in this paper were chosen on the basis of their representativeness and clarity in illustrating various aspects of the selected themes.

## 3 RESULTS

One hundred and ten participants responded to the initial screening survey, of whom 100 met eligibility criteria based on their survey responses. Twenty‐five individuals were contacted for interviews and 17 interviews were completed based on the endpoint of data saturation, determined when no new themes were emerging from interview data (Saunders et al., 2018). Tables 1 and 2 describe the participant demographics.

**Table 1:** Demographics of study participants (*n* = 17)

	*n* (%)
Gender	
Male	7 (41%)
Female	9 (53%)
Other	1 (6%)
Age	
18–25	1 (6%)
25–34	6 (35%)
35–44	4 (24%)
45–54	1 (6%)
55+	5 (29%)
Self‐reported race	
American Indian/Alaska Native	1 (6%)
Asian	1 (6%)
Black or African‐American	2 (12%)
White	11 (65%)
More than one race	2 (12%)
Ethnicity	
Hispanic/Latino	4 (24%)
Not Hispanic/Latino	13 (76%)
Highest level of education completed	
Some college	2 (12%)
Bachelor's degree	7 (41%)
Doctoral or professional degree	8 (47%)
Eye condition	
Retinitis pigmentosa	9 (53%)
Leber congenital amaurosis	8 (47%)
Age of onset	
Birth or infancy	8 (47%)
Childhood	3 (18%)
Adolescence	3 (18%)
Adulthood	3 (18%)

**Table 2:** Selected demographic details by participant

	Condition	Age of onset	Age	Race and ethnicity
P6	RP	Childhood	20s	White, non‐Hispanic
P8	RP	Adulthood	50s	White, Hispanic
P10	RP	Adulthood	30s	White, non‐Hispanic
P17	LCA	Birth or infancy	60s	Black or African‐American, non‐Hispanic
P22	LCA	Birth or infancy	30s	More than one race, Hispanic
P23	LCA	Birth or infancy	20s	More than one race, Hispanic
P27	RP	Childhood	30s	White, non‐Hispanic
P32	LCA	Birth or infancy	30s	Asian, non‐Hispanic
P38	LCA	Birth or infancy	20s	White, non‐Hispanic
P43	LCA	Birth or infancy	40s	White, non‐Hispanic
P47	RP	Teenage years	30s	American Indian/Alaska Native, Hispanic
P48	RP	Adulthood	60s	Black or African‐American, non‐Hispanic
P49	LCA	Birth or infancy	40s	White, non‐Hispanic
P67	RP	Teenage years	50s	Black or African‐American, non‐Hispanic
P76	RP	Childhood	60s	White, non‐Hispanic
P96	RP	Teenage years	80s	White, non‐Hispanic
P102	LCA	Birth or infancy	30s	White, non‐Hispanic

Abbeviations: LCA, Leber congenital amaurosis; RP, retinitis pigmentosa.

### 3.1 Attitudes toward blindness impact views toward hypothetical gene editing

All participants saw potential benefits to gene editing for some medical conditions. Their attitudes toward gene editing for visual conditions; however, diverged in ways that were informed by their lived experiences with and attitudes toward blindness. Some participants felt that blindness was mostly a neutral trait for them, and some felt that it was a positive aspect of their lives; these individuals were less likely to be interested in the idea of somatic gene editing for themselves. Some participants also thought of blindness as something that was integral to their identity. For these participants, that also influenced how they thought about gene editing.

“I understand that to many, visual impairment is a very negative thing to many as something to be cured and something to be fixed, but as someone who lives with this condition, I never really thought of it like that. It was more just it's a part of me and I'm still gonna live my life regardless … I can see why such technology was developed, but I never thought to myself that at the end of a good day or when I'm happy to like, ‘Oh, I'd be so much more happy if only I could see clearly.’ I never once thought that to myself.” (P23, LCA)

“I often think that if my vision loss was completely curable … the person who I've become would be different … So I think I am torn about it.” (P6, RP, childhood‐onset)

Participants who expressed more negative feelings about blindness and its impact on their life, including thinking of it as a medical condition or a defect, were more likely to feel positively about gene editing for blindness and be interested in the idea for themselves.

“Nothing like be[ing] able to do everything the normal human way. When you start having to wear glasses, and those kinds of things … you're not 100%. Whereas [with] genetic therapy, you could get back 100%.” (P96, RP, adult‐onset)

“I can't think of any area where my visual impairment doesn't impact my life. And I will use the word negatively. Again, that doesn't mean coping isn't in place, of course we’re coping. But boy, life sure would be a little easier. Okay, a lot easier.” (P8, RP, adult‐onset)

Many participants fell somewhere in between, in both their attitudes toward blindness and their interest in gene editing. Several thought they would be open to the idea of a gene editing treatment but articulated various considerations they would take into account, such as cost, amount of vision restored, risk, and disruptiveness to their lives.

“The question I think for me would ultimately come down to a lot of details. One is the cost, another is the treatment procedures. The risk, the downside risks as well as the upside potential. And then whether it's something I'd have to do repeatedly, or is it like this one time thing … Because I do have some vision at this point in time, if there's a risk I could lose what I have, that would be a factor … I think if it's a few thousand dollars I would be much more inclined to seriously consider it than if it were a six figure cost. And if we're talking about only a marginal increase in my vision … then I might be much less inclined than if we're talking about a large enough increase … that it would have a meaningful impact on the functional utility of the vision I have.” (P22, LCA)

People who thought of blindness as mostly neutral or having positive aspects often contrasted this with other types of conditions, which they sometimes identified as more worthy targets for gene editing treatment. These participants compared blindness (and often deafness, another sensory disability), which causes no pain or discomfort, is not life‐threatening, and does not inherently limit quality of life, with conditions that have those features. This, too, was often influenced by their personal experiences knowing people with these other conditions.

“I personally wouldn't choose blindness as an early priority for this technology … I would be much more excited about something that could prevent or treat breast cancer, for example, because I’ve lost two family members to breast cancer. So for me I feel like something like that, that could have given them ten or fifteen more years of life would have been really great, or I mean, other life threatening conditions … I would prioritize [that] over something like blindness.” (P102, LCA)

Individuals’ attitudes as to whether they would personally use germline gene editing were mostly consistent with their attitudes toward somatic editing, with those who were interested in curing their own blindness generally also expressing interest in technology to avoid having a blind child and those who were comfortable with their blindness being less interested in it. However, two participants who felt positively about the role of blindness in their own lives and were not strongly interested in treatment still expressed that they would be interested in germline editing to prevent a child from inheriting their condition. These individuals both believed that their personal positive experience with blindness did not guarantee a child would have the same experience.

“I don't know if my kids are going to be as strong as me … I would want them to have that experience like I do, but I don't know if they will … because I don't know if they're going to be as tough as me.” (P47, RP, adolescent‐onset)

### 3.2 Age of onset of blindness impacted views toward hypothetical gene editing

Many participants observed that individuals who were blind from an early age and those who became blind later in life had different attitudes toward blindness, and thus predicted they might have different attitudes toward gene editing.

“[T]here is this argument of … if you could get your vision back would you? And a lot of people will say they wouldn't. I think those are for people who … are born blind. And if you're born blind … you kind of didn't lose anything.” (P8, RP, adult‐onset)

“I think a lot people who have RP, and … their symptoms start later than mine did … they might be people that would really benefit or be interested in [gene editing] because they have a harder time adjusting, or adapting to their vision loss.” (P6, RP, childhood‐onset)

In our sample, generally speaking, those participants who became blind in adolescence or adulthood (*n* = 6) felt more negatively toward being blind and the impacts it had had on their lives than those who had been blind since birth or childhood, and were less likely to think of blindness as a fundamental part of their identity. Some participants with later‐onset, progressive vision loss emphasized the stress of continually adapting to changes in their vision, and three specified that they'd be particularly interested in gene editing to prevent further vision loss, even if they had adjusted to the amount of vision they currently had.

“I have friends who have been blind since birth that have no desire if there were cures or anything like that because this is their life. They've known it, whereas somebody like me, I would really love to at least stop it where it's at. Not to say I can't live as a fully blind person, but if I have the chance, I would love to stop it right where it's at.” (P10, RP, adult‐onset)

People who had been blind from birth or childhood were less likely to be interested in gene editing; several pointed out that they did not feel any sense of loss or that they were missing anything due to their blindness. For many of these participants, the idea of vision might hold some interest as a curiosity, but it was not a high priority in their lives. Some also noted skepticism about the efficacy of sight restoration for adults who had never had vision, and how well they could adapt. However, two participants with congenital‐onset blindness were very interested in gene editing and had sought out information about clinical trials, and two participants with later‐onset RP felt that blindness was a very positive aspect of their lives and had no pressing desire to change it.

In addition to age of onset, degree of visual impairment was raised by some participants as a relevant factor. A couple participants raised the idea that being partially sighted was more challenging than being totally blind, because these individuals did not completely fit into either the blind or sighted world, and that they might benefit more from treatment. On the other hand, one participant with some remaining vision gave that as a reason that she was not interested in gene editing and thought that people who were totally blind would benefit more.

### 3.3 Views about societal impacts of gene editing

#### 3.3.1 Elimination of blindness or other traits

Some participants saw gene editing, particularly germline editing, as a potential means to “eliminate” retinal conditions or other disabilities. Some were troubled by this, especially those who saw blindness as analogous to other identities or traits, and what they saw as a potential for making the world more homogeneous. Diversity, and more specifically ability diversity, including blindness, was seen by these participants as worth preserving. A few saw gene editing for disability as a possible first step on a slippery slope toward eliminating other traits, like skin color or sexual orientation. Three participants specifically drew parallels between gene editing and the eugenics movement.

“… People with LCA have lots of positive traits that may or may not be in some way related to LCA … But I think a diverse society is a good thing, and the idea of trying to create a homogenized society by eliminating differences is a dangerous thing. And I see blindness as a difference rather than as a detriment.” (P17, LCA)

“When I think about this blindness genetic editing, the next thing that pops up for me is, ‘Okay, well … they want to fix the blind. We've got the Foundation Fighting Blindness. Perhaps we should start the Foundation Fighting Blackness, and we should figure out how to make all those poor black people white, and then … this is me basically saying if you're going to suggest that we have to stamp out the blindness, why would you not suggest that we stamp out everything else?” (P49, LCA)

For some, their negative reaction to the idea of eliminating RP or LCA was directly related to the connection they felt with the blind community or people with their particular condition.

“I think on a gut level … I feel connected with the blind community and I feel even more connected with people with LCA … and so I do have a kind of negative feeling about the idea of there not being anyone else born with LCA in kind of a quote‐unquote ideal situation where they could just edit it out of the gene pool entirely … [I]n the Facebook groups that I’m in, especially for LCA … somebody comes in and says I have LCA or my child has LCA … even though I know that the parent usually is not happy about that and I want to be supportive of that parent, but there's a part of me that’s like ‘yay’, like ‘I have a new brother or sister, that’s so cool.’” (P102, LCA)

However, not everyone saw the idea of eliminating blindness or disability as a problem; one participant analogized it to historical efforts to eradicate infectious conditions like polio, and saw the idea of reducing the number of people with disabilities as a generally positive thing. One participant specifically expressed that he did *not* think blindness was analogous to traits like sexuality and skin color, because unlike those traits, it inherently precluded certain activities. Additionally, even some participants troubled by the idea of eliminating blindness spoke more favorably about the idea of eliminating other disabilities and medical conditions, particularly ones that caused pain or impaired quality of life.

“I think for disabilities that are kind of subjectively unpleasant ‐ not just in terms of how you interact with your environment but actually making you feel pain or negative mental states, it makes sense to want to eradicate those kind of disabilities.” (P102, LCA)

#### 3.3.2 Blindness, social barriers, and gene editing

Most participants identified the most negative impacts of blindness in their lives as social, related to discrimination (particularly in employment), social attitudes, and accessibility. Some participants saw gene editing as beneficial specifically because it could get rid of these social barriers for individuals who underwent a treatment, or prevent children who were born without a retinal condition due to germline editing from having to deal with them.

“[A] lot of parents have to fight with their school systems to get appropriate Braille instruction, and you know their kids … have difficult times. They can be discriminated against, they can be bullied, so I think [germline gene editing] would allay a lot of fears for parents and make their lives easier, not to mention the children not having to grow up … being marked as really different.” (P76, RP, adult‐onset)

“To me the main benefit is actually not the vision itself, it's sort of the secondary benefits of circumventing negative social attitudes and discriminatory treatment that people with disabilities experience in society.” (P22, LCA)

However, some expressed frustration at the fact that society found it more desirable or easier to “cure” blindness than to address these barriers.

“[L]et's just face it, the world does not care. It doesn't matter how accessible I would like things to be. The fact is that it is much easier to just remove this thing from the population in a high view than it is to deal with it.” (P49, LCA)

While gene editing could be seen as a way to circumvent social barriers associated with blindness, several participants also expressed concerns about how the availability of gene editing might *exacerbate* social barriers for blind people, particularly those who might be “left behind”––those who could not access gene editing due to medical reasons (including having nongenetic forms of blindness) or financial hardship, or who chose not to have it.

“I just don't want people to think, oh my goodness, don't worry blind people, so what if the unemployment rate amongst blind people is 70% … now with gene editing, all the blind people will be able to see and now they'll be able to be employed … society is going to think, oh no need to make accessibility our prime focus or making accommodations … because there will be no more blind people.” (P47, RP, adolescent‐onset)

The potentially high cost of gene editing was specifically mentioned by many participants as a factor that could exacerbate injustice and disparities.

“[T]he people who are left behind would be the people in the lower socioeconomic echelons … a lot of the impetus for developing technology that blind people can use and the impetus for fighting for laws and fighting for rights has come from people who have an education who have been raised to feel empowered and articulate. And who’ve had families who have been able to push for them. So if those people all disappear, who's going to fight for everybody else? They're going to be forgotten.” (P17, LCA)

Additionally, a few participants voiced concerns about how the availability of gene editing might itself increase stigma toward blindness and blind people and perpetuate the attitude that blindness is something that needs to be “fixed,” rather than an acceptable form of human variation.

“I feel like the downside would be that more people would look at this condition as something to be fixed and if someone had it, they're like, ‘Oh, something's wrong with you.’ And … they're lesser than, because you have this condition.” (P23, LCA)

“So I think the negative impact would be that someone would want to jump to fixing it, when really we just need to be looking more at disabilities as being a part of a person, and not necessarily something that needs fixing.” (P6, RP, childhood‐onset)

A few participants also expressed concern about the way the rhetoric surrounding gene editing and other curative technology, from researchers, families, and the press, can be derogatory to blind people, which could feed into discriminatory attitudes.

“I see a lot of … articles … about like ‘curing blindness’ and … bringing people who are suffering … out of darkness and all these kinds of things, which totally make me want to vomit.” (P38, LCA)

“I was reading … some testimony from … parents of a one year old who had just been diagnosed with LCA … saying things like, we stared at the plain white Braille books in his nursery and cried because he wouldn't be able to enjoy the beautiful print books that we had selected for him. And I love Braille, I get so much joy out of reading Braille … and I just found that really kind of belittling of my own life to be told that I was reading the plain white Braille books … I think the biggest concern I have in the near term is this issue of rhetoric and how policy is made about gene editing … I want to find a way that we can support it that does not belittle life without the technology.” (P102, LCA)

Some participants worried that the idea of a near‐term cure might negatively impact people's adaptation to blindness, including how sighted parents raised their blind children, leading them to put less emphasis on acquiring the skills and education to successfully live life as a blind person.

“I really worry about this this sort of chimera of hope that we're going to fix all these blind people and give them sight and it leads people to go through life waiting for cure rather than living a life. And I really worry because I see it in families and parents a lot … kids who are subjected to surgery after surgery after surgery to try to fix vision or … save some little speck of vision that they've got and they're always going to doctors and recovering from something … and it just seems to be so stultifying, so ultimately emotionally damaging.” (P17, LCA)

In addition, a few participants raised concerns about the messages and expectations that sighted family members might convey to their blind children by seeking out gene editing for them; this concern was drawn from their own experiences.

“Your kid is blind. Why are you trying to worry about fixing it instead of showing them how to be in this world as they are? I remember, my dad always wanted me to be able to see … I think that's a heck of a lot to put on a child, and it's unfair … I just think that when parents put this hope and dream of the genetic gods fixing their child on their child's shoulders, I think that they don't even consciously know that that's what they're doing, but for me that's what they're doing … Are you really not okay with accepting that your child can be who they are and helping them move forward and be a productive, contributing, happy member of society?” (P49, LCA)

#### 3.3.3 Autonomy and consent

The issue of autonomy and consent came up in several different ways related to gene editing. Many participants emphasized how important it was that gene editing be a *choice* or an *option* without coercion. Participants also pointed out that society denying benefits or accommodations to people who chose not to utilize gene editing, either for treatment or for reproduction, could be a form of coercion.

“[P]eople need to have the right to choose, if somebody decides they don't want to undergo this kind of treatment I think it should be their right to do that.” (P76, RP, adult‐onset)

“I think where I feel concerned is somebody deciding that it has to be done. If you don't do this, we're not going to cover you for insurance because you chose, you know what I mean?” (P49, LCA)

Participants varied in their attitudes about parental decision‐making about gene editing for children. Some saw this as equivalent to other decisions we allow parents to make on behalf of their children, while others saw it as taking away the future choices of the child to decide at a later point. Autonomy also came up as a concern in the context of germline gene editing; a few participants were uncomfortable with the idea of a parent choosing traits for a future child without their consent.

“[K]ind of like how the deaf community … have the situation of cochlear implants … and one of the arguments is that … that child doesn't get the choice to be a part of the deaf community, and their parents are making that choice for them. I think this would be something similar.” (P6, RP, childhood‐onset)

“I don't know how I would feel if I knew that I was, for want of a better word, tampered with before I was born and always wondering what was I supposed to be like. I think that it's really important to be able to make that choice yourself as an adult or as a teenager.” (P10, RP, adult‐onset)

Given that many forms of hereditary blindness are recessive, and thus parents of children with these conditions are usually sighted, a few participants also expressed concerns that these parents would be making a decision for their children based on a limited or biased understanding of life as a blind person. They believed this lack of exposure to accurate information about blindness would be a failure of informed consent.

“I think because parents know so little about blindness and generally have never met a blind person who was living successfully they’re awash in ignorance and fear. And they're going to do what the doctors tell them, and doctors are no better informed than parents about reality of life as a blind person. So they're not going to get … what I might consider to be good information.” (P17, LCA)

#### 3.3.4 Views toward enhancement

When presented with the idea of gene editing for enhancement purposes, most participants had negative reactions to the idea, even if they approved of gene editing for other purposes. Objections included the idea of furthering inequality; that it reflected misplaced priorities; and the creation of a never‐ending “arms race” for further ability.

Some participants felt it was straightforward to draw a line between “medical” uses of gene editing and “enhancement.” Others saw the distinction as being less clear, and contingent on the definition of a disability or medical condition, which was not seen by everyone as static or universal, often based on their own experiences.

“I think people in the broader community who see conditions like LCA as negative things, for them they see a difference [between treatment and enhancement]. Whereas I think it's kind of all on a spectrum … I don't see myself as deficient. So I think of adding something to myself as an enhancement rather than as just bringing myself back to the same level as others. I feel like I'm already mostly on the same level of others, so I feel like gaining sight would just be an enhancement.” (P102, LCA)

“Well not being able to run fast isn't really a disability. But you know if most people could run really fast and you couldn't, pretty soon that will start to be seen as a disability.” (P17, LCA)

#### 3.3.5 The drive toward control and perfection

Some participants identified an unease with the idea of gene editing, either for blindness or in the context of enhancement, based on the idea that it reflected or fostered desire for control and a lack of acceptance of people as they are, or could prevent people from accepting the hand they had been dealt. While only one participant explicitly brought up religious objections regarding gene editing of embryos, discomfort with the idea of “playing God” came up several times in the context of germline gene editing and enhancement.

“[W]hen you're editing the genes before they've even … developed, it's like you're kind of playing God in a way. And I don't know, that doesn't sit well with me. That you are kind of predicting and projecting what you want for someone to become.” (P6, RP, childhood onset)

One participant drew on her own lived experience of blindness and the ways it had shaped her life path to explain her unease with the idea of choosing traits for a future child rather than leaving it up to fate:

“I've already lived some of my life and I enjoyed it pretty damn good. And as I said, I love everything about it. And what if we gave that chance to an embryo and we didn't give them RP and maybe their life wasn't wonderful and great and we took away that RP … let's say I was a sighted kid and I never went blind and RP was never an option because somebody treated it and then I went and joined the [military] and I got deployed to go to war, and I didn't come back. And that was my life, and that was it. That would have been a short one. I'm not saying that it would have been, but … what I'm saying is that I don't think it's really truly up to us to decide that.” (P47, RP, adolescent‐onset)

However, some participants saw no conflict between accepting or even embracing blindness in their own lives and curing or preventing it through gene editing.

“I think that science is there to be used, and … whatever challenges we're faced with … should be accepted by each individual, but at the same time, if there is a technology or a scientific solution out there that can improve that, why not use it?” (P43, LCA)

## 4 DISCUSSION

In our study we found that people with RP and LCA held diverse attitudes toward gene editing for visual conditions, influenced by their own views toward and experiences with blindness. Many participants also raised concerns about the social impacts of these technologies that were also drawn from their experiences with the existing social dimensions of blindness. Participants had a unique position informed by their lived experience with which to view the potential benefits and harms of gene editing technology for visual conditions. Many of their perspectives can be contextualized within the broader disability studies literature, which often expresses viewpoints at odds with the narratives of mainstream bioethics (Amundson & Tresky, 2008).

Participants’ beliefs about blindness and gene editing can be partly understood in the context of various “models” of disability. Those who expressed beliefs similar to the medical model, in which an impairment such as blindness is seen as a physical defect that is the proximate cause of hardship for people who have it (Shakespeare, 2006), generally found the idea of treating or preventing blindness to be unproblematic. However, most participants at least partly endorsed views similar to the social model of disability, which states that disability is created by societal factors such as discrimination and access barriers (Shakespeare, 2006). Endorsing the social model did not necessarily predict views toward gene editing: some participants saw a potential negative impact from gene editing due to worsening these social barriers, while others saw it as a way that people could circumvent them. For the latter participants, taking actions that were under their control––for example, treating blindness or avoiding the birth of a blind child––seemed like a pragmatic approach in the face of disabling social barriers that seemed unlikely to change. For the former, there was a concern that, even if some individuals might be able to avoid social barriers as a result of gene editing, discrimination, and access to resources could worsen for those who remained blind.

The affirmative model of disability (Swain & French, 2000), which positions disability as a positive source of individual and community identity, also was reflected in some participants’ views on gene editing for blindness. Those who saw blindness as a positive part of their identity and a source of meaning, value, and community were less interested in gene editing for themselves, and more troubled by the idea of eliminating blindness from the world. Their own experiences with blindness as something that brought unique opportunities to their lives, involved them in enriching communities, and was an inextricable part of themselves and the lives they loved became sources of what has been termed “counter‐eugenic logic”, arguments against the idea that a world without disability would be a desirable one (Garland‐Thomson, 2012).

The finding that individuals with congenital and adult‐onset blindness generally have different attitudes toward their blindness, and thus attitudes toward gene editing, is not unexpected. As many participants pointed out, people who have been blind since a young age generally have no feeling of “loss” and are more likely to be well‐adapted to navigating the world without vision, while those who become blind later in life often face challenges in adjusting. In addition, age of onset has impacts on factors like identity formation, community involvement, and disability self‐efficacy, which in turn have been found to influence attitudes toward curative technologies like gene therapy and selective technologies like prenatal testing (Boardman et al., 2018; Bogart, 2014; Hahn & Belt, 2004).

Some participants endorsed a view about gene editing similar to the “expressivist” argument surrounding prenatal testing and termination for disability, which states that the use of these technologies both expresses and perpetuates negative attitudes toward disability and disabled people (Parens & Asch, 2003). Interestingly, in this study, though many participants compared germline editing to existing technologies like prenatal testing and pre‐implantation genetic diagnosis, the expressivist argument was not limited to reproduction; some participants believed that the use of somatic gene editing as a treatment for visual conditions would also encourage a harmful attitude that blindness was something to be “fixed” or that blind people were lesser than sighted people. A few people extended concern about this from the societal context into parent‐child relationships, and the message that parents might be sending blind children by seeking out a “cure” for them. It has been observed that families that have members with genetic conditions are arenas where the expressivist objection can perhaps be most keenly felt, as parents grapple with what their reproductive decisions mean in relation to their own disability or that of their affected children (Boardman, 2014); this may be true as well for the modified version of the expressivist objection related to treatment presented here. A softer version of the expressivist argument was made by a few participants who did not raise the concern that the existence of gene editing technology perpetuated these ideas, but did think that the way we talk about it could.

In general, what bioethicists often position as a crucial distinction between germline and somatic editing was not as prevalent in the views of the participants in this study. For the most part, those who thought of blindness as a medical condition or negative trait welcomed both germline and somatic applications as methods of alleviating it. Meanwhile, those who felt more positive about blindness and had concerns about the societal impacts of gene editing for blindness usually had concerns about both types of gene editing, because they were similar in the aspects that were viewed as most salient in terms of their impact on blind people: having the potential to reduce the population of blind people, and in being representative of and perpetuating particular views toward blindness. Perhaps for similar reasons, most participants did not bring up a distinction between prenatal testing and selective termination and germline editing, with the exception of one participant who opposed termination more strongly on religious grounds but ultimately was still uncomfortable with germline editing because he felt people might still terminate if editing was unsuccessful. Additionally, concerns about autonomy and informed consent and how gene editing would impact identity formation were raised in the context of both germline editing and somatic editing for children, although the timing of intervention was seen as important by some.

Participants’ views toward enhancement were mostly negative, similar to findings in the general public (Funk, Kennedy, & Sciupac, 2016; McCaughey et al., 2016). For many participants, the idea of using gene editing for enhancement was clearly different than using it for blindness or other disabilities. However, for a few participants, who did not consider blindness to be a medical condition or negative trait, their lived experience led them to conclude the distinction between “enhancement” and “treatment” was subjective. Scully and Rehman‐Sutter (2001) have described how making the enhancement/treatment distinction requires the creation of a “normal” standard, which has implications for those who are therefore positioned as “abnormal.” For those who consider their blindness to be a type of normal, categorizing sight‐restoration as inherently distinct from “enhancement” contradicts their experience of blindness as part of the spectrum of human variation, and of themselves as being on essentially equal footing with sighted people.

Several participants described unease about gene editing and what it reflected and conveyed in ways that centered around ideas about control and acceptance. Political philosopher Michael Sandel has articulated an argument against genetic enhancement predicated on the notion that it reflects a harmful desire for mastery and control, and “misses the part of freedom that consists in a persisting negotiation with the given” (Sandel, 2007). While Sandel limits his argument to enhancement and specifically notes his approval of genetic modification for “disease,” similar ideas were expressed by several participants in relation to blindness. Several participants described the benefits of negotiating the hand that they had been dealt with their blindness, and worried about what was implied when parents could not accept a child being blind. Just as some did not see “enhancement” and “treatment” as completely distinct because of their view that blindness was part of the spectrum of human difference, these participants saw a desire for control in relation to blindness as not essentially different from trying to control other traits.

Many participants contrasted blindness with other conditions that they thought of as more severe, and thus potentially more important targets for gene editing. The existence of a mental “hierarchy of impairments” has been described among both disabled and nondisabled individuals, but the order of this hierarchy is far from universal (Deal, 2003). For instance, while several participants in this study specifically mentioned cancer as worse than blindness, the general public has been found to rank blindness as more severe than cancer in terms of perceived impact on quality of life (American Foundation for the Blind, 2007). Quality of life, while mentioned by many participants as a deciding factor in whether gene editing should be pursued for a given condition, is subjective, and people with chronic health conditions and disabilities have been consistently found to rank their own quality of life higher than predicted by family members, professionals, and the public (Albrecht & Devlieger, 1999; Crocker, Smith, & Skevington, 2015). Thus, it is clear that the only reliable way to learn how individuals with a particular disability view that disability, and what their attitude might be toward treating or curing it, is to ask them––and to take their answer seriously––which researchers and bioethicists have often failed to do (Goering, 2008). The devaluing of the accounts of disabled people regarding their own lives is a form of “epistemic injustice” that has profound implications for decision‐making that affects them (Scully, 2018). The views of people with inherited retinal conditions toward gene editing are likely to be different than those of people with other conditions, since the disability experiences of those with sensory impairments like blindness differ in many ways from those of people with other types of disabilities, such as intellectual disabilities, mobility impairments, and chronic illness. Factors such as stigma, accessibility, the existence of community, physical pain, and other aspects of the lived experience of disability can vary greatly between disabilities (as well as within them) and are likely to influence views. Thus, it is crucial that people with any type of disability or health condition for which gene editing research is being conducted be included in dialogue about it.

## 5 LIMITATIONS

Given the lack of prior literature in this area, we conducted an exploratory qualitative study, which was intended to gain a broad understanding of key issues, rather than to draw systematic conclusions about the views of the general population of people with inherited retinal conditions or blindness. Recruitment through the listserv of the NFB, an organization with a philosophy emphasizing the capabilities of blind people, likely biased the sample toward participants with greater involvement in the blind community and more positive attitudes toward blindness, although not all participants were actively involved in or endorsed the philosophy of the organization. The vast majority of respondents to the screening survey, and thus interview participants, were also highly educated (88% with a Bachelor's degree or higher), which is not representative of the general population of blind people in the United States (~16%; Erickson, Lee, & von Schrader, 2017). In addition, the generalizability of these findings made within an American context to other cultures may be limited; attitudes toward disability and the social context in which it operates, vary greatly across cultures. Factors such as cost of treatment and the possibility of coercion may also be conceived of quite differently in countries with different types of healthcare systems. Despite these limitations, participants expressed a wide range of views that can be used as a starting point to explore the attitudes in people with inherited retinal conditions toward gene editing. Further research on larger samples is necessary to determine the prevalence of these various attitudes in the larger population of people with inherited retinal conditions and blind people more generally.

## 6 CONCLUSION

Participants in this study, even when they expressed concerns about gene editing, still believed it had potential benefits and thought research, at least for some medical applications, should continue. But many also raised concerns about how the clinical use of gene editing could impact blind people and society, informed by their own experiential knowledge, including some important near‐term considerations for scientists, policymakers, medical professionals. It is important to discuss and promote gene editing technology in a way that is not derogatory toward blind people and their capabilities, and is conscious of the fact that many individuals may consider their blindness to be an important and valuable part of who they are. Freedom of choice and informed consent––including accurate, unbiased information about the lives of blind people for sighted parents considering gene editing for their children––are vital. And societal investment in accessibility and inclusion must not be impacted by the prospect of a “cure” or treatment for certain forms of blindness, nor should access to resources be impacted by an individual's choice to utilize or not utilize gene editing. The voices of those affected by genetic conditions and disabilities must be included, and prioritized, in societal decision‐making about gene editing.

## ENDNOTE

1 Dunn and Burcaw (2013) define disability identity as “entail[ing] a positive sense of self, feelings of connection to, or solidarity with, the disability community.”
