# Supplementary material for: Attitudes of people with inherited retinal conditions toward gene editing technology
Source: Mol Genet Genomic Med. 2019 Jun 12;7(7):e00803. doi: 10.1002/mgg3.803 (PMC6625087; doi:10.1002/mgg3.803)
Supplement: Supplementary file 1 [file MGG3-7-e00803-s001.docx]

**Interview guide**

This interview is designed to learn about your experiences with (RP/LCA/blindness/low vision), as well as your thoughts, feelings, and attitudes about a new technology that is being developed, called gene editing.

First, I have some questions about you and your (RP/LCA/blindness/low vision).

1. **Tell me about your experience of having (RP/LCA/blindness/low vision).**

Probes: When did you develop (RP/LCA/blindness/low vision)?

What type of vision, if any, do you have now?

What positive aspects, if any, have there been to having (RP/LCA/blindness/low vision)?

What negative aspects, if any, have there been to having (RP/LCA/blindness/low vision)?

To what extent are you involved in blind or disability communities? What has that been like?

1. **Some people who have (RP/LCA/blindness/low vision) consider it to be part of their identity. What do you think about this? Is this something that you consider to be true for you?**

Now I’m going to explain a little bit about a technology called gene editing, and ask you some questions about your thoughts and feelings about it. You may have also heard this technology called CRISPR.

1. **What, if anything, have you heard about this technology?**

Our body is made up of millions of cells, and almost every cell in our body contains genes. Genes hold the instructions for how our body grows, develops and functions. Different versions of genes are what cause Leber congenital amaurosis and retinitis pigmentosa. Gene *editing* is a technology that allows scientists to cut out one version of a gene and replace it with another version, for example, replacing a version of a gene that causes LCA or RP with one that does not.

There are two main categories of gene editing that scientists are working on. The first category I’m going to ask questions about is called somatic gene editing, where scientists edit the genes in just one part of the body when someone has already been born. For instance, scientists might edit the genes in someone’s eye to stop the progression of low vision. This technology is just starting to be researched for some forms of vision loss.

1. **What do you think of this technology in general?**

Probes: What concerns do you have, if any, about how it might be used?

What benefits, if any, do you think there could be to this technology?

1. **Do you think this technology should be available for people with medical problems or disabilities? Why or why not?**

Probes: What types of conditions do you think it should be available for?

Do you think this technology should be available for people with (RP/LCA/blindness/low vision)? Why or why not?

Do you think there is a difference between this technology being available for kids and for adults?

1. **If this technology were available, would you want to use it for your (RP/LCA/blindness/low vision)? Why or why not?**
2. **What do you think the impacts on society would be of having this technology available? What about the impacts on people with (RP/LCA/blindness/low vision)?**

Next, I’m going to ask you about a second type of gene editing scientists are working on, called germline editing. In this type of technology, scientists would edit the genes in all of the cells of an embryo at the beginning of its development. For example, they could edit genes so that the baby that grew from an embryo that would otherwise have RP or LCA would never develop it. Not only would this baby not develop RP or LCA, he or she would also not be able to pass down RP or LCA to his or her children. This technology is not available now, but might be in the future.

1. **What do you think of this use of gene editing technology?**

Probes: What concerns do you have, if any, about how it might be used?

What benefits, if any, do you think there could be to this use of gene editing?

How do you think this use of technology is different from using gene editing in an adult?

1. **If this technology were available, would you want to use it if you were having children? Why or why not?**

Now, I am going to ask you about another way that people might use gene editing technology. Some people think that gene editing technology might be used for “enhancement” of people that would otherwise be average. For example, it could potentially be used to give a person better athletic ability, or to give a sighted person who would otherwise have average vision better than average vision.

1. **What do you think about this possible use of gene editing technology?**
2. **What do you think is the difference, if any, between using gene editing for enhancement and using it for a medical condition or disability?**
3. **Do you have any other thoughts you would like to add?**
